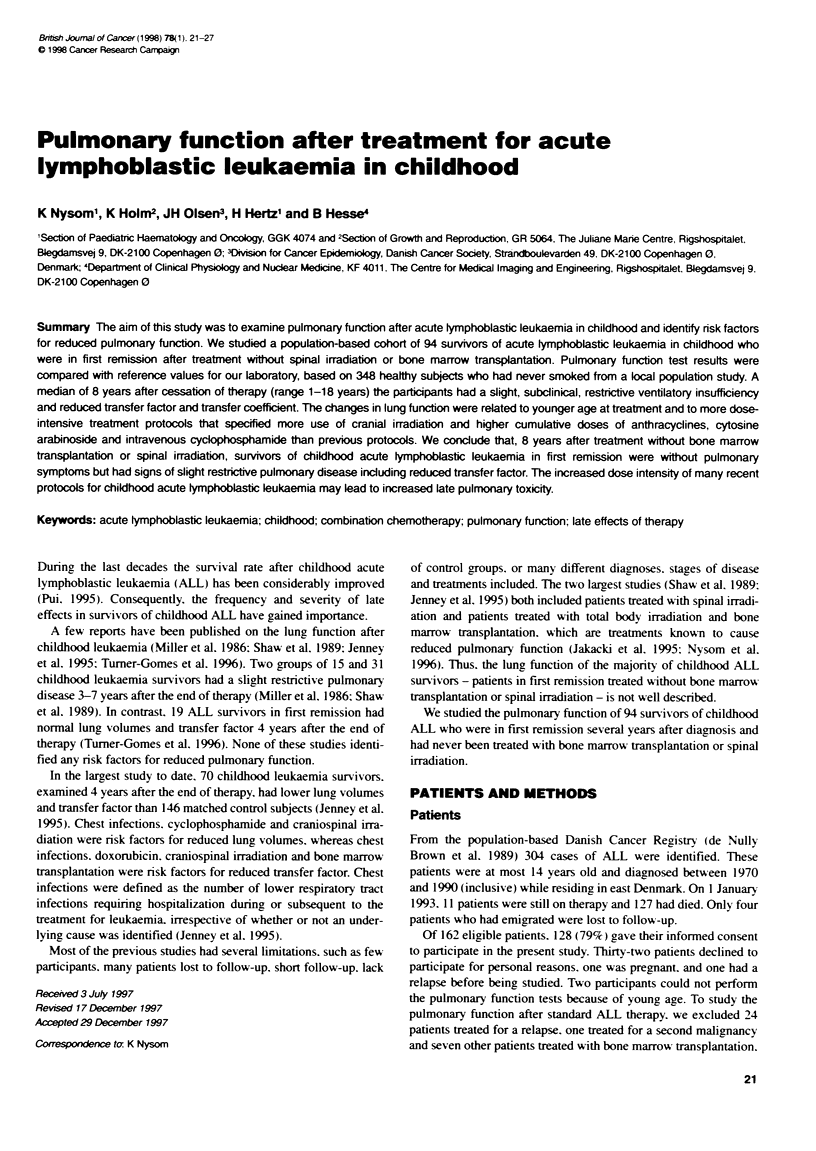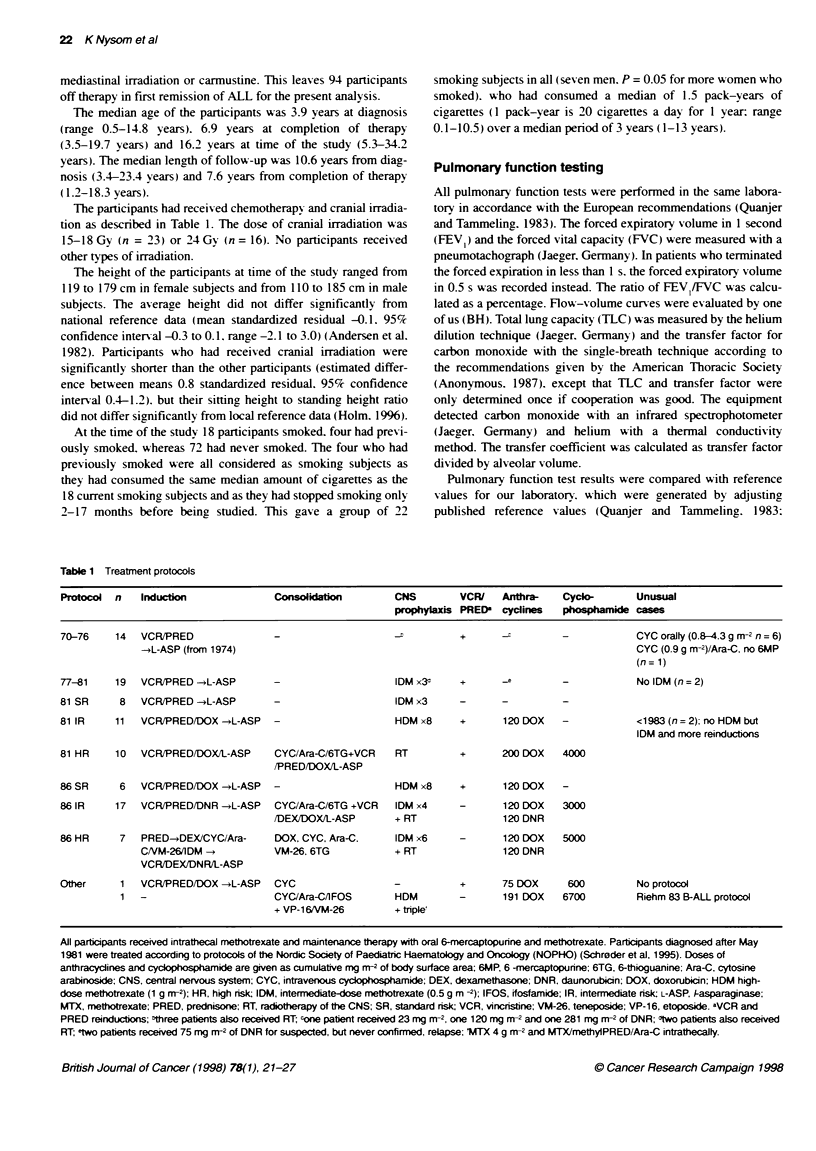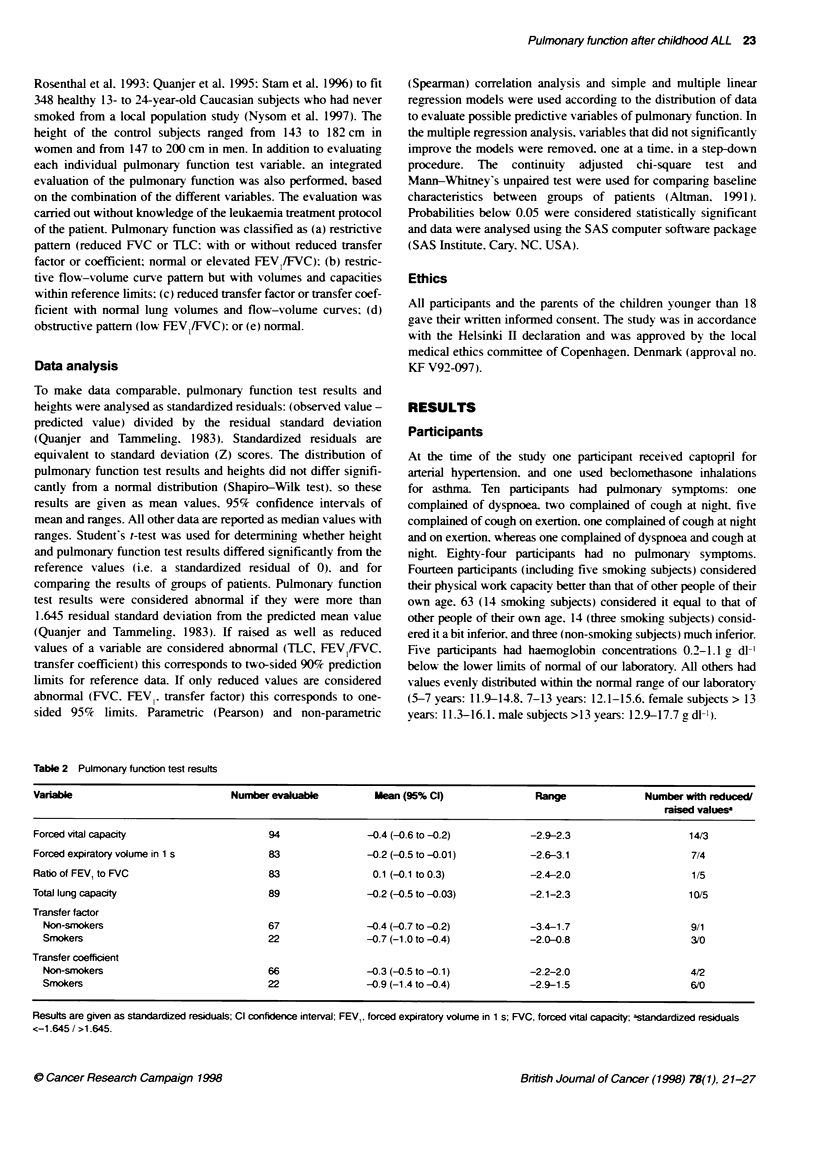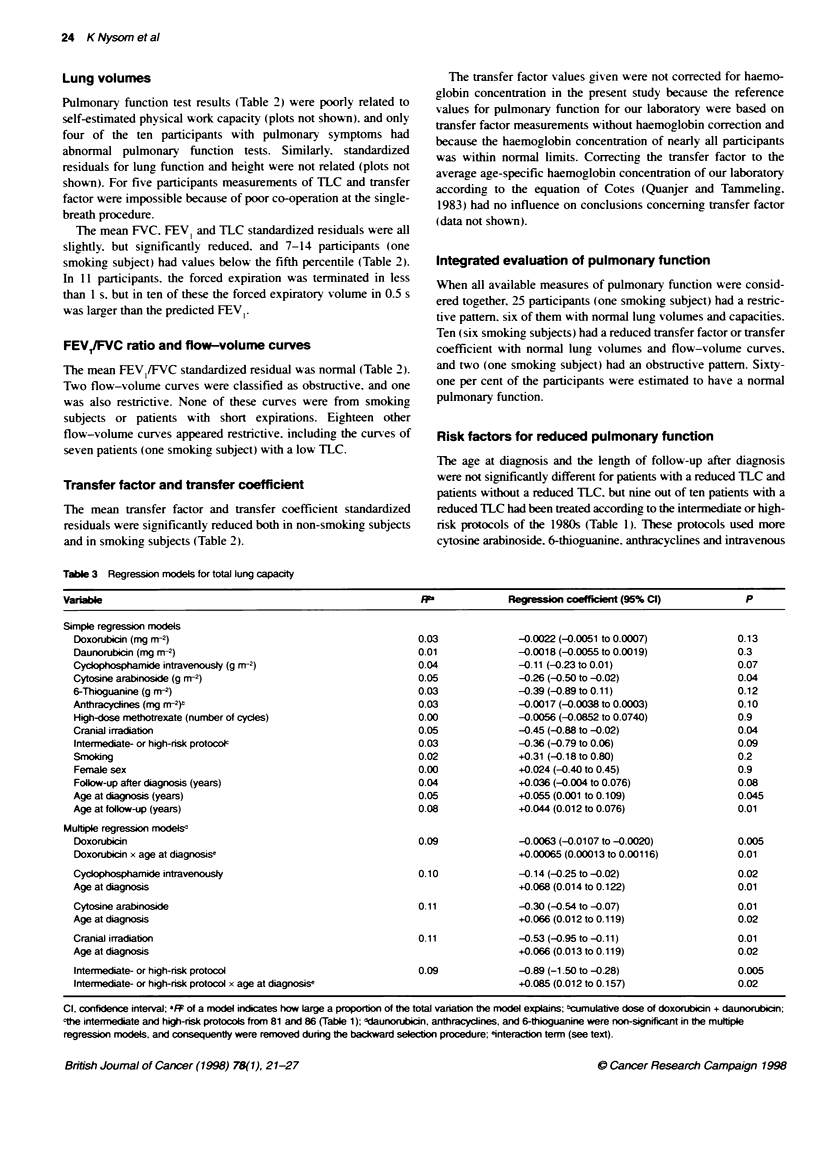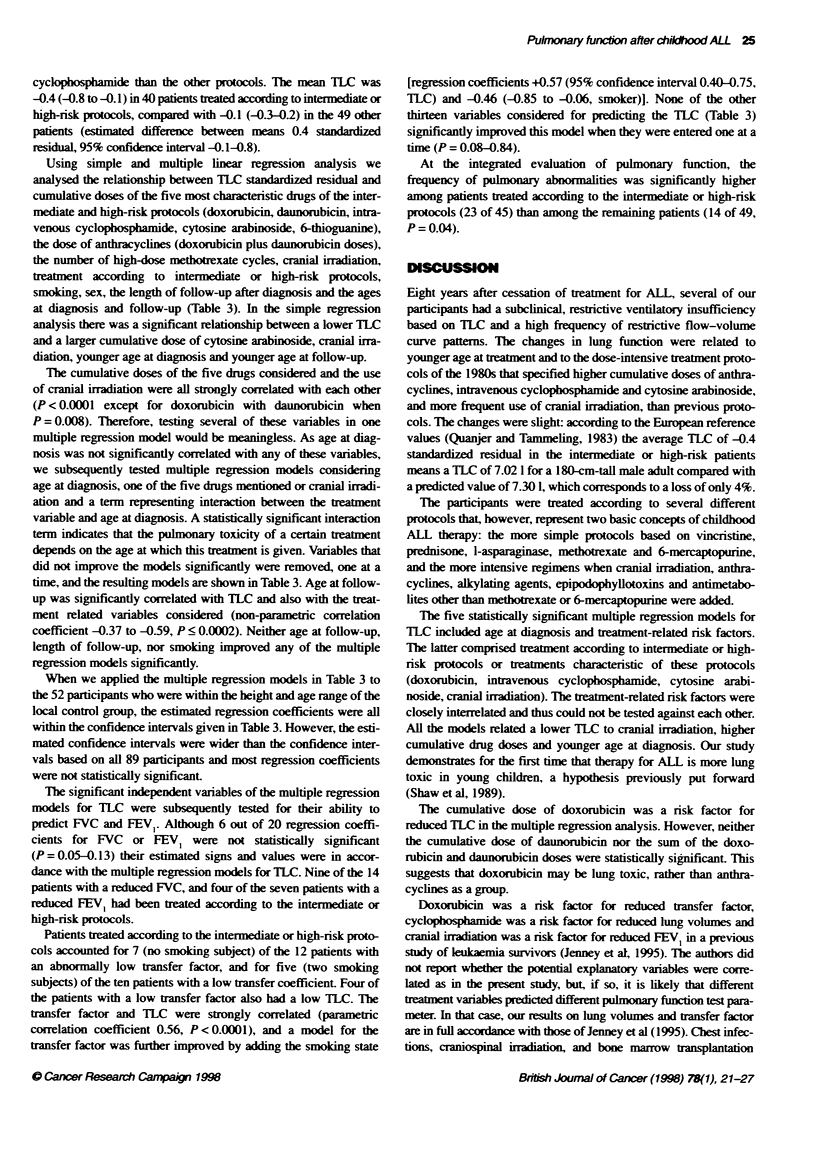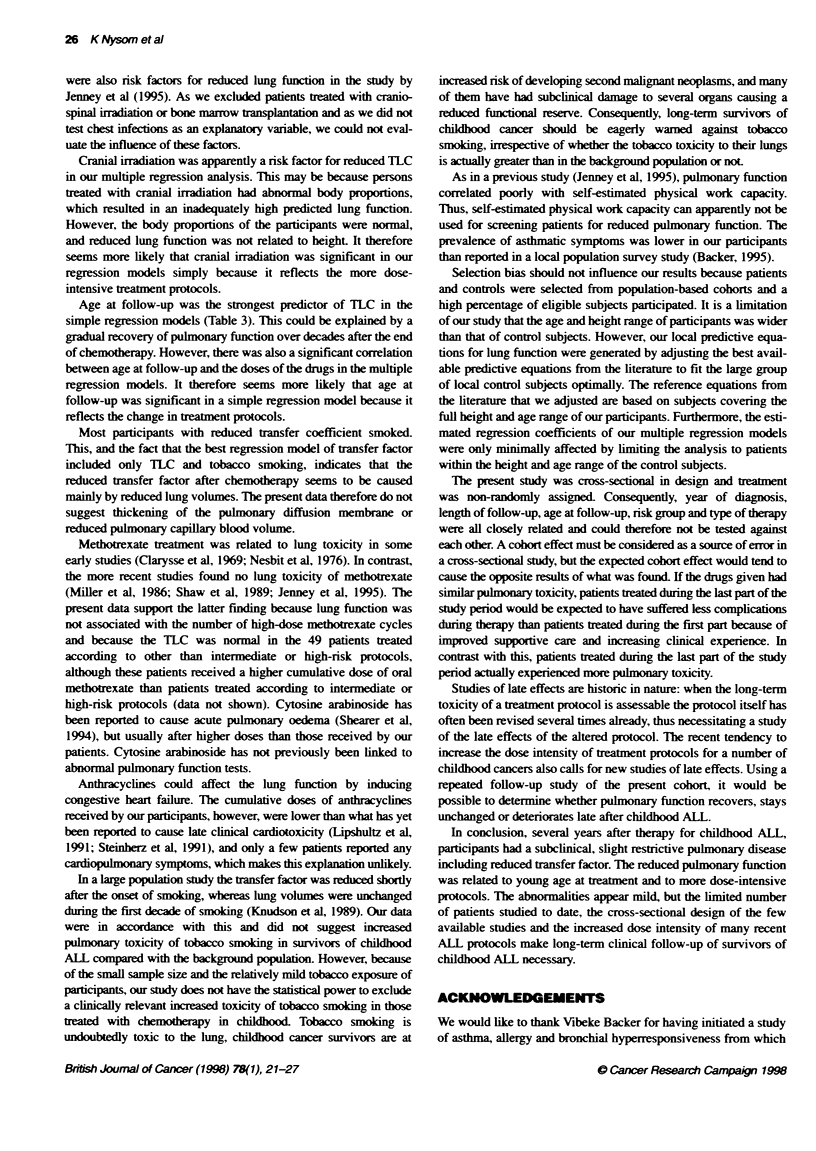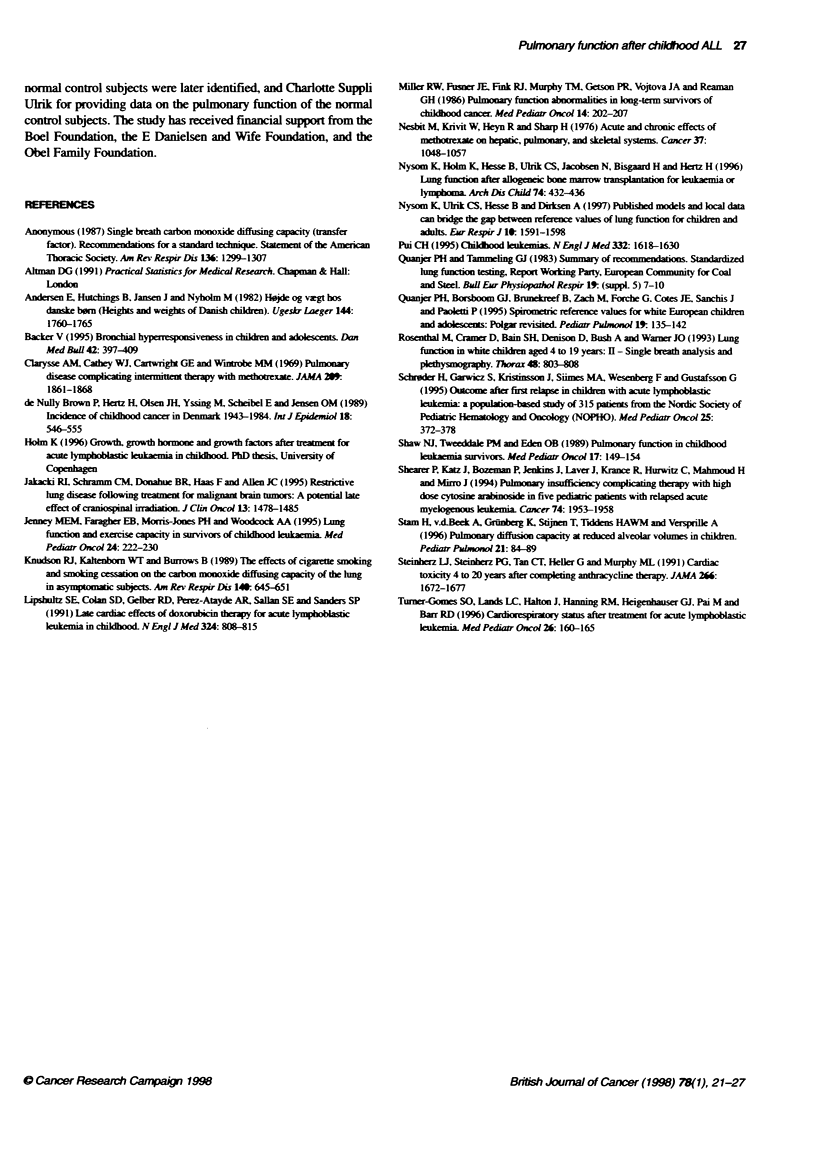# Pulmonary function after treatment for acute lymphoblastic leukaemia in childhood.

**DOI:** 10.1038/bjc.1998.436

**Published:** 1998-07

**Authors:** K. Nysom, K. Holm, J. H. Olsen, H. Hertz, B. Hesse

**Affiliations:** Section of Paediatric Haematology and Oncology, GGK 4074, The Juliane Marie Centre, Rigshospitalet, Copenhagen.

## Abstract

The aim of this study was to examine pulmonary function after acute lymphoblastic leukaemia in childhood and identify risk factors for reduced pulmonary function. We studied a population-based cohort of 94 survivors of acute lymphoblastic leukaemia in childhood who were in first remission after treatment without spinal irradiation or bone marrow transplantation. Pulmonary function test results were compared with reference values for our laboratory, based on 348 healthy subjects who had never smoked from a local population study. A median of 8 years after cessation of therapy (range 1-18 years) the participants had a slight, subclinical, restrictive ventilatory insufficiency and reduced transfer factor and transfer coefficient. The changes in lung function were related to younger age at treatment and to more dose-intensive treatment protocols that specified more use of cranial irradiation and higher cumulative doses of anthracyclines, cytosine arabinoside and intravenous cyclophosphamide than previous protocols. We conclude that, 8 years after treatment without bone marrow transplantation or spinal irradiation, survivors of childhood acute lymphoblastic leukaemia in first remission were without pulmonary symptoms but had signs of slight restrictive pulmonary disease including reduced transfer factor. The increased dose intensity of many recent protocols for childhood acute lymphoblastic leukaemia may lead to increased late pulmonary toxicity.


British Journal of Cancer (1998) 78(1). 21–27
© 1998 Cancer Research Campaign

### Pulmonary function after treatment for acute lymphoblastic leukaemia in childhood

**K Nysom[1], K Holm[2], JH Olsen[3], H Hertz[1] and B Hesse[4]**

[1]Section of Paediatric Haematology and Oncology, GGK 4074 and [2]Section of Growth and Reproduction, GR 5064, The Juliane Marie Centre, Rigshospitalet, Blegdamsvej 9, DK-2100 Copenhagen Ø; [3]Division for Cancer Epidemiology, Danish Cancer Society, Strandboulevarden 49, DK-2100 Copenhagen Ø, Denmark; [4]Department of Clinical Physiology and Nuclear Medicine, KF 4011, The Centre for Medical Imaging and Engineering, Rigshospitalet, Blegdamsvej 9, DK-2100 Copenhagen Ø

**Summary** The aim of this study was to examine pulmonary function after acute lymphoblastic leukaemia in childhood and identify risk factors for reduced pulmonary function. We studied a population-based cohort of 94 survivors of acute lymphoblastic leukaemia in childhood who were in first remission after treatment without spinal irradiation or bone marrow transplantation. Pulmonary function test results were compared with reference values for our laboratory, based on 348 healthy subjects who had never smoked from a local population study. A median of 8 years after cessation of therapy (range 1–18 years) the participants had a slight, subclinical, restrictive ventilatory insufficiency and reduced transfer factor and transfer coefficient. The changes in lung function were related to younger age at treatment and to more dose-intensive treatment protocols that specified more use of cranial irradiation and higher cumulative doses of anthracyclines, cytosine arabinoside and intravenous cyclophosphamide than previous protocols. We conclude that, 8 years after treatment without bone marrow transplantation or spinal irradiation, survivors of childhood acute lymphoblastic leukaemia in first remission were without pulmonary symptoms but had signs of slight restrictive pulmonary disease including reduced transfer factor. The increased dose intensity of many recent protocols for childhood acute lymphoblastic leukaemia may lead to increased late pulmonary toxicity.

**Keywords:** acute lymphoblastic leukaemia; childhood; combination chemotherapy; pulmonary function; late effects of therapy

During the last decades the survival rate after childhood acute lymphoblastic leukaemia (ALL) has been considerably improved (Pui, 1995). Consequently, the frequency and severity of late effects in survivors of childhood ALL have gained importance.

A few reports have been published on the lung function after childhood leukaemia (Miller et al, 1986; Shaw et al, 1989; Jenney et al, 1995; Turner-Gomes et al, 1996). Two groups of 15 and 31 childhood leukaemia survivors had a slight restrictive pulmonary disease 3–7 years after the end of therapy (Miller et al, 1986; Shaw et al, 1989). In contrast, 19 ALL survivors in first remission had normal lung volumes and transfer factor 4 years after the end of therapy (Turner-Gomes et al, 1996). None of these studies identified any risk factors for reduced pulmonary function.

In the largest study to date, 70 childhood leukaemia survivors, examined 4 years after the end of therapy, had lower lung volumes and transfer factor than 146 matched control subjects (Jenney et al, 1995). Chest infections, cyclophosphamide and craniospinal irradiation were risk factors for reduced lung volumes, whereas chest infections, doxorubicin, craniospinal irradiation and bone marrow transplantation were risk factors for reduced transfer factor. Chest infections were defined as the number of lower respiratory tract infections requiring hospitalization during or subsequent to the treatment for leukaemia, irrespective of whether or not an underlying cause was identified (Jenney et al, 1995).

Most of the previous studies had several limitations, such as few participants, many patients lost to follow-up, short follow-up, lack of control groups, or many different diagnoses, stages of disease and treatments included. The two largest studies (Shaw et al, 1989; Jenney et al, 1995) both included patients treated with spinal irradiation and patients treated with total body irradiation and bone marrow transplantation, which are treatments known to cause reduced pulmonary function (Jakacki et al, 1995; Nysom et al, 1996). Thus, the lung function of the majority of childhood ALL survivors – patients in first remission treated without bone marrow transplantation or spinal irradiation – is not well described.

We studied the pulmonary function of 94 survivors of childhood ALL who were in first remission several years after diagnosis and had never been treated with bone marrow transplantation or spinal irradiation.

*Received 3 July 1997*
*Revised 17 December 1997*
*Accepted 29 December 1997*

*Correspondence to:* K Nysom

### PATIENTS AND METHODS

### Patients

From the population-based Danish Cancer Registry (de Nully Brown et al, 1989) 304 cases of ALL were identified. These patients were at most 14 years old and diagnosed between 1970 and 1990 (inclusive) while residing in east Denmark. On 1 January 1993, 11 patients were still on therapy and 127 had died. Only four patients who had emigrated were lost to follow-up.

Of 162 eligible patients, 128 (79%) gave their informed consent to participate in the present study. Thirty-two patients declined to participate for personal reasons, one was pregnant, and one had a relapse before being studied. Two participants could not perform the pulmonary function tests because of young age. To study the pulmonary function after standard ALL therapy, we excluded 24 patients treated for a relapse, one treated for a second malignancy and seven other patients treated with bone marrow transplantation.

mediastinal irradiation or carmustine. This leaves 94 participants off therapy in first remission of ALL for the present analysis.

The median age of the participants was 3.9 years at diagnosis (range 0.5–14.8 years), 6.9 years at completion of therapy (3.5–19.7 years) and 16.2 years at time of the study (5.3–34.2 years). The median length of follow-up was 10.6 years from diagnosis (3.4–23.4 years) and 7.6 years from completion of therapy (1.2–18.3 years).

The participants had received chemotherapy and cranial irradiation as described in Table 1. The dose of cranial irradiation was 15–18 Gy ($n$ = 23) or 24 Gy ($n$ = 16). No participants received other types of irradiation.

The height of the participants at time of the study ranged from 119 to 179 cm in female subjects and from 110 to 185 cm in male subjects. The average height did not differ significantly from national reference data (mean standardized residual –0.1, 95% confidence interval –0.3 to 0.1, range –2.1 to 3.0) (Andersen et al, 1982). Participants who had received cranial irradiation were significantly shorter than the other participants (estimated difference between means 0.8 standardized residual, 95% confidence interval 0.4–1.2), but their sitting height to standing height ratio did not differ significantly from local reference data (Holm, 1996).

At the time of the study 18 participants smoked, four had previously smoked, whereas 72 had never smoked. The four who had previously smoked were all considered as smoking subjects as they had consumed the same median amount of cigarettes as the 18 current smoking subjects and as they had stopped smoking only 2–17 months before being studied. This gave a group of 22 smoking subjects in all (seven men, $P$ = 0.05 for more women who smoked), who had consumed a median of 1.5 pack–years of cigarettes (1 pack–year is 20 cigarettes a day for 1 year; range 0.1–10.5) over a median period of 3 years (1–13 years).

### Pulmonary function testing

All pulmonary function tests were performed in the same laboratory in accordance with the European recommendations (Quanjer and Tammeling, 1983). The forced expiratory volume in 1 second ($FEV_1$) and the forced vital capacity (FVC) were measured with a pneumotachograph (Jaeger, Germany). In patients who terminated the forced expiration in less than 1 s, the forced expiratory volume in 0.5 s was recorded instead. The ratio of $FEV_1$/FVC was calculated as a percentage. Flow–volume curves were evaluated by one of us (BH). Total lung capacity (TLC) was measured by the helium dilution technique (Jaeger, Germany) and the transfer factor for carbon monoxide with the single-breath technique according to the recommendations given by the American Thoracic Society (Anonymous, 1987), except that TLC and transfer factor were only determined once if cooperation was good. The equipment detected carbon monoxide with an infrared spectrophotometer (Jaeger, Germany) and helium with a thermal conductivity method. The transfer coefficient was calculated as transfer factor divided by alveolar volume.

Pulmonary function test results were compared with reference values for our laboratory, which were generated by adjusting published reference values (Quanjer and Tammeling, 1983;

**Table 1** Treatment protocols

| Protocol | $n$ | Induction | Consolidation | CNS prophylaxis | VCR/ PRED[a] | Anthra-cyclines | Cyclo-phosphamide | Unusual cases |
|---|---|---|---|---|---|---|---|---|
| 70–76 | 14 | VCR/PRED →L-ASP (from 1974) | – | –[b] | + | –[c] | – | CYC orally (0.8–4.3 g m$^{-2}$ $n$ = 6) CYC (0.9 g m$^{-2}$)/Ara-C, no 6MP ($n$ = 1) |
| 77–81 | 19 | VCR/PRED →L-ASP | – | IDM ×3[d] | + | –[e] | – | No IDM ($n$ = 2) |
| 81 SR | 8 | VCR/PRED →L-ASP | – | IDM ×3 | – | – | – | |
| 81 IR | 11 | VCR/PRED/DOX →L-ASP | – | HDM ×8 | + | 120 DOX | – | <1983 ($n$ = 2); no HDM but IDM and more reinductions |
| 81 HR | 10 | VCR/PRED/DOX/L-ASP | CYC/Ara-C/6TG+VCR /PRED/DOX/L-ASP | RT | + | 200 DOX | 4000 | |
| 86 SR | 6 | VCR/PRED/DOX →L-ASP | – | HDM ×8 | + | 120 DOX | – | |
| 86 IR | 17 | VCR/PRED/DNR →L-ASP | CYC/Ara-C/6TG +VCR /DEX/DOX/L-ASP | IDM ×4 + RT | – | 120 DOX 120 DNR | 3000 | |
| 86 HR | 7 | PRED→DEX/CYC/Ara-C/VM-26/IDM → VCR/DEX/DNR/L-ASP | DOX, CYC, Ara-C, VM-26, 6TG | IDM ×6 + RT | – | 120 DOX 120 DNR | 5000 | |
| Other | 1 | VCR/PRED/DOX →L-ASP | CYC | – | + | 75 DOX | 600 | No protocol |
| | 1 | – | CYC/Ara-C/IFOS + VP-16/VM-26 | HDM + triple[f] | – | 191 DOX | 6700 | Riehm 83 B-ALL protocol |

All participants received intrathecal methotrexate and maintenance therapy with oral 6-mercaptopurine and methotrexate. Participants diagnosed after May 1981 were treated according to protocols of the Nordic Society of Paediatric Haematology and Oncology (NOPHO) (Schrøder et al, 1995). Doses of anthracyclines and cyclophosphamide are given as cumulative mg m$^{-2}$ of body surface area; 6MP, 6 -mercaptopurine; 6TG, 6-thioguanine; Ara-C, cytosine arabinoside; CNS, central nervous system; CYC, intravenous cyclophosphamide; DEX, dexamethasone; DNR, daunorubicin; DOX, doxorubicin; HDM high-dose methotrexate (1 g m$^{-2}$); HR, high risk; IDM, intermediate-dose methotrexate (0.5 g m $^{-2}$); IFOS, ifosfamide; IR, intermediate risk; L-ASP, L-asparaginase; MTX, methotrexate; PRED, prednisone; RT, radiotherapy of the CNS; SR, standard risk; VCR, vincristine; VM-26, teneposide; VP-16, etoposide. [a]VCR and PRED reinductions; [b]three patients also received RT; [c]one patient received 23 mg m$^{-2}$, one 120 mg m$^{-2}$ and one 281 mg m$^{-2}$ of DNR; [d]two patients also received RT; [e]two patients received 75 mg m$^{-2}$ of DNR for suspected, but never confirmed, relapse; [f]MTX 4 g m$^{-2}$ and MTX/methylPRED/Ara-C intrathecally.

© Cancer Research Campaign 1998

Rosenthal et al. 1993; Quanjer et al. 1995; Stam et al. 1996) to fit 348 healthy 13- to 24-year-old Caucasian subjects who had never smoked from a local population study (Nysom et al. 1997). The height of the control subjects ranged from 143 to 182 cm in women and from 147 to 200 cm in men. In addition to evaluating each individual pulmonary function test variable, an integrated evaluation of the pulmonary function was also performed, based on the combination of the different variables. The evaluation was carried out without knowledge of the leukaemia treatment protocol of the patient. Pulmonary function was classified as (a) restrictive pattern (reduced FVC or TLC; with or without reduced transfer factor or coefficient; normal or elevated $FEV_1$/FVC); (b) restrictive flow–volume curve pattern but with volumes and capacities within reference limits; (c) reduced transfer factor or transfer coefficient with normal lung volumes and flow–volume curves; (d) obstructive pattern (low $FEV_1$/FVC); or (e) normal.

### Data analysis

To make data comparable, pulmonary function test results and heights were analysed as standardized residuals: (observed value – predicted value) divided by the residual standard deviation (Quanjer and Tammeling, 1983). Standardized residuals are equivalent to standard deviation (Z) scores. The distribution of pulmonary function test results and heights did not differ significantly from a normal distribution (Shapiro–Wilk test), so these results are given as mean values, 95% confidence intervals of mean and ranges. All other data are reported as median values with ranges. Student's *t*-test was used for determining whether height and pulmonary function test results differed significantly from the reference values (i.e. a standardized residual of 0), and for comparing the results of groups of patients. Pulmonary function test results were considered abnormal if they were more than 1.645 residual standard deviation from the predicted mean value (Quanjer and Tammeling, 1983). If raised as well as reduced values of a variable are considered abnormal (TLC, $FEV_1$/FVC, transfer coefficient) this corresponds to two-sided 90% prediction limits for reference data. If only reduced values are considered abnormal (FVC, $FEV_1$, transfer factor) this corresponds to one-sided 95% limits. Parametric (Pearson) and non-parametric (Spearman) correlation analysis and simple and multiple linear regression models were used according to the distribution of data to evaluate possible predictive variables of pulmonary function. In the multiple regression analysis, variables that did not significantly improve the models were removed, one at a time, in a step-down procedure. The continuity adjusted chi-square test and Mann–Whitney's unpaired test were used for comparing baseline characteristics between groups of patients (Altman, 1991). Probabilities below 0.05 were considered statistically significant and data were analysed using the SAS computer software package (SAS Institute, Cary, NC, USA).

### Ethics

All participants and the parents of the children younger than 18 gave their written informed consent. The study was in accordance with the Helsinki II declaration and was approved by the local medical ethics committee of Copenhagen, Denmark (approval no. KF V92-097).

### RESULTS

### Participants

At the time of the study one participant received captopril for arterial hypertension, and one used beclomethasone inhalations for asthma. Ten participants had pulmonary symptoms: one complained of dyspnoea, two complained of cough at night, five complained of cough on exertion, one complained of cough at night and on exertion, whereas one complained of dyspnoea and cough at night. Eighty-four participants had no pulmonary symptoms. Fourteen participants (including five smoking subjects) considered their physical work capacity better than that of other people of their own age, 63 (14 smoking subjects) considered it equal to that of other people of their own age, 14 (three smoking subjects) considered it a bit inferior, and three (non-smoking subjects) much inferior. Five participants had haemoglobin concentrations 0.2–1.1 g $dl^{-1}$ below the lower limits of normal of our laboratory. All others had values evenly distributed within the normal range of our laboratory (5–7 years: 11.9–14.8, 7–13 years: 12.1–15.6, female subjects > 13 years: 11.3–16.1, male subjects >13 years: 12.9–17.7 g $dl^{-1}$).

**Table 2** Pulmonary function test results

| Variable | Number evaluable | Mean (95% CI) | Range | Number with reduced/ raised values[a] |
|---|---|---|---|---|
| Forced vital capacity | 94 | –0.4 (–0.6 to –0.2) | -2.9–2.3 | 14/3 |
| Forced expiratory volume in 1 s | 83 | –0.2 (–0.5 to –0.01) | -2.6–3.1 | 7/4 |
| Ratio of $FEV_1$ to FVC | 83 | 0.1 (–0.1 to 0.3) | -2.4–2.0 | 1/5 |
| Total lung capacity | 89 | –0.2 (–0.5 to –0.03) | -2.1–2.3 | 10/5 |
| Transfer factor | | | | |
| Non-smokers | 67 | –0.4 (–0.7 to –0.2) | -3.4–1.7 | 9/1 |
| Smokers | 22 | –0.7 (–1.0 to –0.4) | -2.0–0.8 | 3/0 |
| Transfer coefficient | | | | |
| Non-smokers | 66 | –0.3 (–0.5 to –0.1) | -2.2–2.0 | 4/2 |
| Smokers | 22 | –0.9 (–1.4 to –0.4) | -2.9–1.5 | 6/0 |

Results are given as standardized residuals; CI confidence interval; $FEV_1$, forced expiratory volume in 1 s; FVC, forced vital capacity; [a]standardized residuals <–1.645 / >1.645.

© Cancer Research Campaign 1998

### Lung volumes

Pulmonary function test results (Table 2) were poorly related to self-estimated physical work capacity (plots not shown), and only four of the ten participants with pulmonary symptoms had abnormal pulmonary function tests. Similarly, standardized residuals for lung function and height were not related (plots not shown). For five participants measurements of TLC and transfer factor were impossible because of poor co-operation at the single-breath procedure.

The mean FVC, $FEV_1$ and TLC standardized residuals were all slightly, but significantly reduced, and 7–14 participants (one smoking subject) had values below the fifth percentile (Table 2). In 11 participants, the forced expiration was terminated in less than 1 s, but in ten of these the forced expiratory volume in 0.5 s was larger than the predicted $FEV_1$.

### $FEV_1$/FVC ratio and flow–volume curves

The mean $FEV_1$/FVC standardized residual was normal (Table 2). Two flow–volume curves were classified as obstructive, and one was also restrictive. None of these curves were from smoking subjects or patients with short expirations. Eighteen other flow–volume curves appeared restrictive, including the curves of seven patients (one smoking subject) with a low TLC.

### Transfer factor and transfer coefficient

The mean transfer factor and transfer coefficient standardized residuals were significantly reduced both in non-smoking subjects and in smoking subjects (Table 2).

The transfer factor values given were not corrected for haemoglobin concentration in the present study because the reference values for pulmonary function for our laboratory were based on transfer factor measurements without haemoglobin correction and because the haemoglobin concentration of nearly all participants was within normal limits. Correcting the transfer factor to the average age-specific haemoglobin concentration of our laboratory according to the equation of Cotes (Quanjer and Tammeling, 1983) had no influence on conclusions concerning transfer factor (data not shown).

### Integrated evaluation of pulmonary function

When all available measures of pulmonary function were considered together, 25 participants (one smoking subject) had a restrictive pattern, six of them with normal lung volumes and capacities. Ten (six smoking subjects) had a reduced transfer factor or transfer coefficient with normal lung volumes and flow–volume curves, and two (one smoking subject) had an obstructive pattern. Sixty-one per cent of the participants were estimated to have a normal pulmonary function.

### Risk factors for reduced pulmonary function

The age at diagnosis and the length of follow-up after diagnosis were not significantly different for patients with a reduced TLC and patients without a reduced TLC, but nine out of ten patients with a reduced TLC had been treated according to the intermediate or high-risk protocols of the 1980s (Table 1). These protocols used more cytosine arabinoside, 6-thioguanine, anthracyclines and intravenous

**Table 3** Regression models for total lung capacity

| Variable | $R^2$[a] | Regression coefficient (95% CI) | P |
|---|---|---|---|
| Simple regression models | | | |
| Doxorubicin (mg $m^{-2}$) | 0.03 | −0.0022 (−0.0051 to 0.0007) | 0.13 |
| Daunorubicin (mg $m^{-2}$) | 0.01 | −0.0018 (−0.0055 to 0.0019) | 0.3 |
| Cyclophosphamide intravenously (g $m^{-2}$) | 0.04 | −0.11 (−0.23 to 0.01) | 0.07 |
| Cytosine arabinoside (g $m^{-2}$) | 0.05 | −0.26 (−0.50 to −0.02) | 0.04 |
| 6-Thioguanine (g $m^{-2}$) | 0.03 | −0.39 (−0.89 to 0.11) | 0.12 |
| Anthracyclines (mg $m^{-2}$)[b] | 0.03 | −0.0017 (−0.0038 to 0.0003) | 0.10 |
| High-dose methotrexate (number of cycles) | 0.00 | −0.0056 (−0.0852 to 0.0740) | 0.9 |
| Cranial irradiation | 0.05 | −0.45 (−0.88 to −0.02) | 0.04 |
| Intermediate- or high-risk protocol[c] | 0.03 | −0.36 (−0.79 to 0.06) | 0.09 |
| Smoking | 0.02 | +0.31 (−0.18 to 0.80) | 0.2 |
| Female sex | 0.00 | +0.024 (−0.40 to 0.45) | 0.9 |
| Follow-up after diagnosis (years) | 0.04 | +0.036 (−0.004 to 0.076) | 0.08 |
| Age at diagnosis (years) | 0.05 | +0.055 (0.001 to 0.109) | 0.045 |
| Age at follow-up (years) | 0.08 | +0.044 (0.012 to 0.076) | 0.01 |
| Multiple regression models[d] | | | |
| Doxorubicin | 0.09 | −0.0063 (−0.0107 to −0.0020) | 0.005 |
| Doxorubicin × age at diagnosis[e] | | +0.00065 (0.00013 to 0.00116) | 0.01 |
| Cyclophosphamide intravenously | 0.10 | −0.14 (−0.25 to −0.02) | 0.02 |
| Age at diagnosis | | +0.068 (0.014 to 0.122) | 0.01 |
| Cytosine arabinoside | 0.11 | −0.30 (−0.54 to −0.07) | 0.01 |
| Age at diagnosis | | +0.066 (0.012 to 0.119) | 0.02 |
| Cranial irradiation | 0.11 | −0.53 (−0.95 to −0.11) | 0.01 |
| Age at diagnosis | | +0.066 (0.013 to 0.119) | 0.02 |
| Intermediate- or high-risk protocol | 0.09 | −0.89 (−1.50 to −0.28) | 0.005 |
| Intermediate- or high-risk protocol × age at diagnosis[e] | | +0.085 (0.012 to 0.157) | 0.02 |

CI, confidence interval; [a]$R^2$ of a model indicates how large a proportion of the total variation the model explains; [b]cumulative dose of doxorubicin + daunorubicin; [c]the intermediate and high-risk protocols from 81 and 86 (Table 1); [d]daunorubicin, anthracyclines, and 6-thioguanine were non-significant in the multiple regression models, and consequently were removed during the backward selection procedure; [e]interaction term (see text).

© Cancer Research Campaign 1998

cyclophosphamide than the other protocols. The mean TLC was –0.4 (–0.8 to –0.1) in 40 patients treated according to intermediate or high-risk protocols, compared with –0.1 (–0.3–0.2) in the 49 other patients (estimated difference between means 0.4 standardized residual, 95% confidence interval –0.1–0.8).

Using simple and multiple linear regression analysis we analysed the relationship between TLC standardized residual and cumulative doses of the five most characteristic drugs of the intermediate and high-risk protocols (doxorubicin, daunorubicin, intravenous cyclophosphamide, cytosine arabinoside, 6-thioguanine), the dose of anthracyclines (doxorubicin plus daunorubicin doses), the number of high-dose methotrexate cycles, cranial irradiation, treatment according to intermediate or high-risk protocols, smoking, sex, the length of follow-up after diagnosis and the ages at diagnosis and follow-up (Table 3). In the simple regression analysis there was a significant relationship between a lower TLC and a larger cumulative dose of cytosine arabinoside, cranial irradiation, younger age at diagnosis and younger age at follow-up.

The cumulative doses of the five drugs considered and the use of cranial irradiation were all strongly correlated with each other ($P < 0.0001$ except for doxorubicin with daunorubicin when $P = 0.008$). Therefore, testing several of these variables in one multiple regression model would be meaningless. As age at diagnosis was not significantly correlated with any of these variables, we subsequently tested multiple regression models considering age at diagnosis, one of the five drugs mentioned or cranial irradiation and a term representing interaction between the treatment variable and age at diagnosis. A statistically significant interaction term indicates that the pulmonary toxicity of a certain treatment depends on the age at which this treatment is given. Variables that did not improve the models significantly were removed, one at a time, and the resulting models are shown in Table 3. Age at follow-up was significantly correlated with TLC and also with the treatment related variables considered (non-parametric correlation coefficient –0.37 to –0.59, $P \leq 0.0002$). Neither age at follow-up, length of follow-up, nor smoking improved any of the multiple regression models significantly.

When we applied the multiple regression models in Table 3 to the 52 participants who were within the height and age range of the local control group, the estimated regression coefficients were all within the confidence intervals given in Table 3. However, the estimated confidence intervals were wider than the confidence intervals based on all 89 participants and most regression coefficients were not statistically significant.

The significant independent variables of the multiple regression models for TLC were subsequently tested for their ability to predict FVC and $FEV_1$. Although 6 out of 20 regression coefficients for FVC or $FEV_1$ were not statistically significant ($P = 0.05–0.13$) their estimated signs and values were in accordance with the multiple regression models for TLC. Nine of the 14 patients with a reduced FVC, and four of the seven patients with a reduced $FEV_1$ had been treated according to the intermediate or high-risk protocols.

Patients treated according to the intermediate or high-risk protocols accounted for 7 (no smoking subject) of the 12 patients with an abnormally low transfer factor, and for five (two smoking subjects) of the ten patients with a low transfer coefficient. Four of the patients with a low transfer factor also had a low TLC. The transfer factor and TLC were strongly correlated (parametric correlation coefficient 0.56, $P < 0.0001$), and a model for the transfer factor was further improved by adding the smoking state [regression coefficients +0.57 (95% confidence interval 0.40–0.75, TLC) and –0.46 (–0.85 to –0.06, smoker)]. None of the other thirteen variables considered for predicting the TLC (Table 3) significantly improved this model when they were entered one at a time ($P = 0.08–0.84$).

At the integrated evaluation of pulmonary function, the frequency of pulmonary abnormalities was significantly higher among patients treated according to the intermediate or high-risk protocols (23 of 45) than among the remaining patients (14 of 49, $P = 0.04$).

### DISCUSSION

Eight years after cessation of treatment for ALL, several of our participants had a subclinical, restrictive ventilatory insufficiency based on TLC and a high frequency of restrictive flow–volume curve patterns. The changes in lung function were related to younger age at treatment and to the dose-intensive treatment protocols of the 1980s that specified higher cumulative doses of anthracyclines, intravenous cyclophosphamide and cytosine arabinoside, and more frequent use of cranial irradiation, than previous protocols. The changes were slight: according to the European reference values (Quanjer and Tammeling, 1983) the average TLC of –0.4 standardized residual in the intermediate or high-risk patients means a TLC of 7.02 l for a 180-cm-tall male adult compared with a predicted value of 7.30 l, which corresponds to a loss of only 4%.

The participants were treated according to several different protocols that, however, represent two basic concepts of childhood ALL therapy: the more simple protocols based on vincristine, prednisone, l-asparaginase, methotrexate and 6-mercaptopurine, and the more intensive regimens when cranial irradiation, anthracyclines, alkylating agents, epipodophyllotoxins and antimetabolites other than methotrexate or 6-mercaptopurine were added.

The five statistically significant multiple regression models for TLC included age at diagnosis and treatment-related risk factors. The latter comprised treatment according to intermediate or high-risk protocols or treatments characteristic of these protocols (doxorubicin, intravenous cyclophosphamide, cytosine arabinoside, cranial irradiation). The treatment-related risk factors were closely interrelated and thus could not be tested against each other. All the models related a lower TLC to cranial irradiation, higher cumulative drug doses and younger age at diagnosis. Our study demonstrates for the first time that therapy for ALL is more lung toxic in young children, a hypothesis previously put forward (Shaw et al, 1989).

The cumulative dose of doxorubicin was a risk factor for reduced TLC in the multiple regression analysis. However, neither the cumulative dose of daunorubicin nor the sum of the doxorubicin and daunorubicin doses were statistically significant. This suggests that doxorubicin may be lung toxic, rather than anthracyclines as a group.

Doxorubicin was a risk factor for reduced transfer factor, cyclophosphamide was a risk factor for reduced lung volumes and cranial irradiation was a risk factor for reduced $FEV_1$ in a previous study of leukaemia survivors (Jenney et al, 1995). The authors did not report whether the potential explanatory variables were correlated as in the present study, but, if so, it is likely that different treatment variables predicted different pulmonary function test parameter. In that case, our results on lung volumes and transfer factor are in full accordance with those of Jenney et al (1995). Chest infections, craniospinal irradiation, and bone marrow transplantation

© Cancer Research Campaign 1998

were also risk factors for reduced lung function in the study by Jenney et al (1995). As we excluded patients treated with craniospinal irradiation or bone marrow transplantation and as we did not test chest infections as an explanatory variable, we could not evaluate the influence of these factors.

Cranial irradiation was apparently a risk factor for reduced TLC in our multiple regression analysis. This may be because persons treated with cranial irradiation had abnormal body proportions, which resulted in an inadequately high predicted lung function. However, the body proportions of the participants were normal, and reduced lung function was not related to height. It therefore seems more likely that cranial irradiation was significant in our regression models simply because it reflects the more dose-intensive treatment protocols.

Age at follow-up was the strongest predictor of TLC in the simple regression models (Table 3). This could be explained by a gradual recovery of pulmonary function over decades after the end of chemotherapy. However, there was also a significant correlation between age at follow-up and the doses of the drugs in the multiple regression models. It therefore seems more likely that age at follow-up was significant in a simple regression model because it reflects the change in treatment protocols.

Most participants with reduced transfer coefficient smoked. This, and the fact that the best regression model of transfer factor included only TLC and tobacco smoking, indicates that the reduced transfer factor after chemotherapy seems to be caused mainly by reduced lung volumes. The present data therefore do not suggest thickening of the pulmonary diffusion membrane or reduced pulmonary capillary blood volume.

Methotrexate treatment was related to lung toxicity in some early studies (Clarysse et al, 1969; Nesbit et al, 1976). In contrast, the more recent studies found no lung toxicity of methotrexate (Miller et al, 1986; Shaw et al, 1989; Jenney et al, 1995). The present data support the latter finding because lung function was not associated with the number of high-dose methotrexate cycles and because the TLC was normal in the 49 patients treated according to other than intermediate or high-risk protocols, although these patients received a higher cumulative dose of oral methotrexate than patients treated according to intermediate or high-risk protocols (data not shown). Cytosine arabinoside has been reported to cause acute pulmonary oedema (Shearer et al, 1994), but usually after higher doses than those received by our patients. Cytosine arabinoside has not previously been linked to abnormal pulmonary function tests.

Anthracyclines could affect the lung function by inducing congestive heart failure. The cumulative doses of anthracyclines received by our participants, however, were lower than what has yet been reported to cause late clinical cardiotoxicity (Lipshultz et al, 1991; Steinherz et al, 1991), and only a few patients reported any cardiopulmonary symptoms, which makes this explanation unlikely.

In a large population study the transfer factor was reduced shortly after the onset of smoking, whereas lung volumes were unchanged during the first decade of smoking (Knudson et al, 1989). Our data were in accordance with this and did not suggest increased pulmonary toxicity of tobacco smoking in survivors of childhood ALL compared with the background population. However, because of the small sample size and the relatively mild tobacco exposure of participants, our study does not have the statistical power to exclude a clinically relevant increased toxicity of tobacco smoking in those treated with chemotherapy in childhood. Tobacco smoking is undoubtedly toxic to the lung, childhood cancer survivors are at increased risk of developing second malignant neoplasms, and many of them have had subclinical damage to several organs causing a reduced functional reserve. Consequently, long-term survivors of childhood cancer should be eagerly warned against tobacco smoking, irrespective of whether the tobacco toxicity to their lungs is actually greater than in the background population or not.

As in a previous study (Jenney et al, 1995), pulmonary function correlated poorly with self-estimated physical work capacity. Thus, self-estimated physical work capacity can apparently not be used for screening patients for reduced pulmonary function. The prevalence of asthmatic symptoms was lower in our participants than reported in a local population survey study (Backer, 1995).

Selection bias should not influence our results because patients and controls were selected from population-based cohorts and a high percentage of eligible subjects participated. It is a limitation of our study that the age and height range of participants was wider than that of control subjects. However, our local predictive equations for lung function were generated by adjusting the best available predictive equations from the literature to fit the large group of local control subjects optimally. The reference equations from the literature that we adjusted are based on subjects covering the full height and age range of our participants. Furthermore, the estimated regression coefficients of our multiple regression models were only minimally affected by limiting the analysis to patients within the height and age range of the control subjects.

The present study was cross-sectional in design and treatment was non-randomly assigned. Consequently, year of diagnosis, length of follow-up, age at follow-up, risk group and type of therapy were all closely related and could therefore not be tested against each other. A cohort effect must be considered as a source of error in a cross-sectional study, but the expected cohort effect would tend to cause the opposite results of what was found. If the drugs given had similar pulmonary toxicity, patients treated during the last part of the study period would be expected to have suffered less complications during therapy than patients treated during the first part because of improved supportive care and increasing clinical experience. In contrast with this, patients treated during the last part of the study period actually experienced more pulmonary toxicity.

Studies of late effects are historic in nature: when the long-term toxicity of a treatment protocol is assessable the protocol itself has often been revised several times already, thus necessitating a study of the late effects of the altered protocol. The recent tendency to increase the dose intensity of treatment protocols for a number of childhood cancers also calls for new studies of late effects. Using a repeated follow-up study of the present cohort, it would be possible to determine whether pulmonary function recovers, stays unchanged or deteriorates late after childhood ALL.

In conclusion, several years after therapy for childhood ALL, participants had a subclinical, slight restrictive pulmonary disease including reduced transfer factor. The reduced pulmonary function was related to young age at treatment and to more dose-intensive protocols. The abnormalities appear mild, but the limited number of patients studied to date, the cross-sectional design of the few available studies and the increased dose intensity of many recent ALL protocols make long-term clinical follow-up of survivors of childhood ALL necessary.

### ACKNOWLEDGEMENTS

We would like to thank Vibeke Backer for having initiated a study of asthma, allergy and bronchial hyperresponsiveness from which

© Cancer Research Campaign 1998

normal control subjects were later identified, and Charlotte Suppli Ulrik for providing data on the pulmonary function of the normal control subjects. The study has received financial support from the Boel Foundation, the E Danielsen and Wife Foundation, and the Obel Family Foundation.

### REFERENCES

Anonymous (1987) Single breath carbon monoxide diffusing capacity (transfer factor). Recommendations for a standard technique. Statement of the American Thoracic Society. *Am Rev Respir Dis* **136**: 1299–1307

Altman DG (1991) *Practical Statistics for Medical Research*. Chapman & Hall: London

Andersen E, Hutchings B, Jansen J and Nyholm M (1982) Højde og vægt hos danske børn (Heights and weights of Danish children). *Ugeskr Laeger* **144**: 1760–1765

Backer V (1995) Bronchial hyperresponsiveness in children and adolescents. *Dan Med Bull* **42**: 397–409

Clarysse AM, Cathey WJ, Cartwright GE and Wintrobe MM (1969) Pulmonary disease complicating intermittent therapy with methotrexate. *JAMA* **209**: 1861–1868

de Nully Brown P, Hertz H, Olsen JH, Yssing M, Scheibel E and Jensen OM (1989) Incidence of childhood cancer in Denmark 1943–1984. *Int J Epidemiol* **18**: 546–555

Holm K (1996) Growth, growth hormone and growth factors after treatment for acute lymphoblastic leukaemia in childhood. PhD thesis, University of Copenhagen

Jakacki RI, Schramm CM, Donahue BR, Haas F and Allen JC (1995) Restrictive lung disease following treatment for malignant brain tumors: A potential late effect of craniospinal irradiation. *J Clin Oncol* **13**: 1478–1485

Jenney MEM, Faragher EB, Morris-Jones PH and Woodcock AA (1995) Lung function and exercise capacity in survivors of childhood leukaemia. *Med Pediatr Oncol* **24**: 222–230

Knudson RJ, Kaltenborn WT and Burrows B (1989) The effects of cigarette smoking and smoking cessation on the carbon monoxide diffusing capacity of the lung in asymptomatic subjects. *Am Rev Respir Dis* **140**: 645–651

Lipshultz SE, Colan SD, Gelber RD, Perez-Atayde AR, Sallan SE and Sanders SP (1991) Late cardiac effects of doxorubicin therapy for acute lymphoblastic leukemia in childhood. *N Engl J Med* **324**: 808–815

Miller RW, Fusner JE, Fink RJ, Murphy TM, Getson PR, Vojtova JA and Reaman GH (1986) Pulmonary function abnormalities in long-term survivors of childhood cancer. *Med Pediatr Oncol* **14**: 202–207

Nesbit M, Krivit W, Heyn R and Sharp H (1976) Acute and chronic effects of methotrexate on hepatic, pulmonary, and skeletal systems. *Cancer* **37**: 1048–1057

Nysom K, Holm K, Hesse B, Ulrik CS, Jacobsen N, Bisgaard H and Hertz H (1996) Lung function after allogeneic bone marrow transplantation for leukaemia or lymphoma. *Arch Dis Child* **74**: 432–436

Nysom K, Ulrik CS, Hesse B and Dirksen A (1997) Published models and local data can bridge the gap between reference values of lung function for children and adults. *Eur Respir J* **10**: 1591–1598

Pui CH (1995) Childhood leukemias. *N Engl J Med* **332**: 1618–1630

Quanjer PH and Tammeling GJ (1983) Summary of recommendations. Standardized lung function testing, Report Working Party, European Community for Coal and Steel. *Bull Eur Physiopathol Respir* **19**: (suppl. 5) 7–10

Quanjer PH, Borsboom GJ, Brunekreef B, Zach M, Forche G, Cotes JE, Sanchis J and Paoletti P (1995) Spirometric reference values for white European children and adolescents: Polgar revisited. *Pediatr Pulmonol* **19**: 135–142

Rosenthal M, Cramer D, Bain SH, Denison D, Bush A and Warner JO (1993) Lung function in white children aged 4 to 19 years: II – Single breath analysis and plethysmography. *Thorax* **48**: 803–808

Schrøder H, Garwicz S, Kristinsson J, Siimes MA, Wesenberg F and Gustafsson G (1995) Outcome after first relapse in children with acute lymphoblastic leukemia: a population-based study of 315 patients from the Nordic Society of Pediatric Hematology and Oncology (NOPHO). *Med Pediatr Oncol* **25**: 372–378

Shaw NJ, Tweeddale PM and Eden OB (1989) Pulmonary function in childhood leukaemia survivors. *Med Pediatr Oncol* **17**: 149–154

Shearer P, Katz J, Bozeman P, Jenkins J, Laver J, Krance R, Hurwitz C, Mahmoud H and Mirro J (1994) Pulmonary insufficiency complicating therapy with high dose cytosine arabinoside in five pediatric patients with relapsed acute myelogenous leukemia. *Cancer* **74**: 1953–1958

Stam H, v.d.Beek A, Grünberg K, Stijnen T, Tiddens HAWM and Versprille A (1996) Pulmonary diffusion capacity at reduced alveolar volumes in children. *Pediatr Pulmonol* **21**: 84–89

Steinherz LJ, Steinherz PG, Tan CT, Heller G and Murphy ML (1991) Cardiac toxicity 4 to 20 years after completing anthracycline therapy. *JAMA* **266**: 1672–1677

Turner-Gomes SO, Lands LC, Halton J, Hanning RM, Heigenhauser GJ, Pai M and Barr RD (1996) Cardiorespiratory status after treatment for acute lymphoblastic leukemia. *Med Pediatr Oncol* **26**: 160–165

© Cancer Research Campaign 1998